# Supplementary material for: Towards unified AI-driven fracture mechanics: the extended deep energy method (XDEM)
Source: Nat Commun. 2026 Aug 15;17:8492. doi: 10.1038/s41467-026-76748-1 (PMC13484471; doi:10.1038/s41467-026-76748-1)
Supplement: Supplementary file 3 [file 41467_2026_76748_MOESM3_ESM.zip › pic/Results/Stress_intensity_factor/Mode2/a05_k2_comparison.pdf]

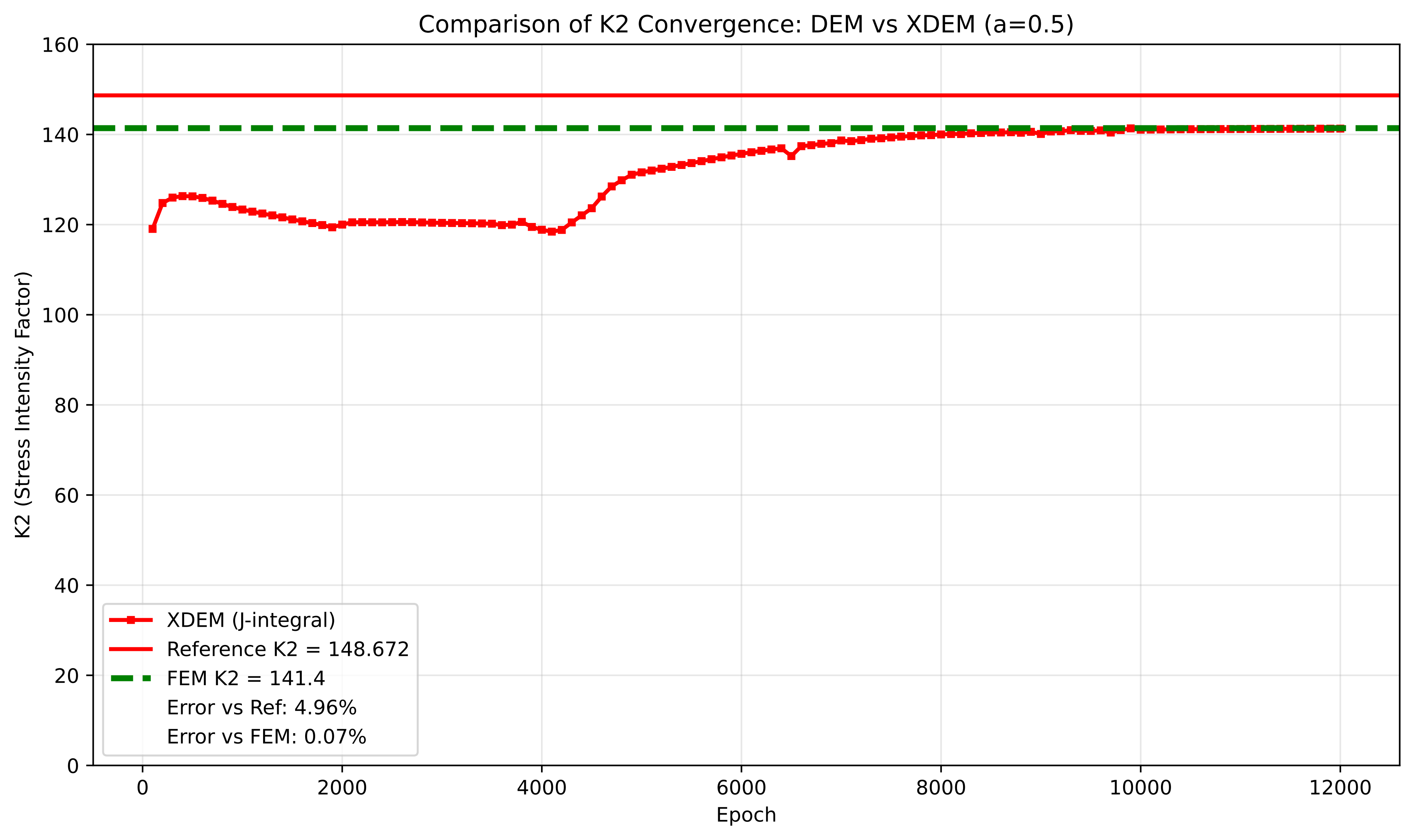

Comparison of K2 Convergence: DEM vs XDEM (a=0.5)
K2 (Stress Intensity Factor)
Epoch
0
20
40
60
80
100
120
140
160
0
2000
4000
6000
8000
10000
12000
XDEM (J-integral)
Reference K2 = 148.672
FEM K2 = 141.4
Error vs Ref: 4.96%
Error vs FEM: 0.07%
